# Vulvitis Infantalis

**Published:** 1880-08

**Authors:** 


Vulvitis Infantalis.—Vulvitis of children generally occurs as a consequence of cold and is liable to recur. It is generally easily cured, and of its treatment it would be useless to say anything, so well is it given in systematic lectures and text books. I have seen it as often among the rich as the poor, and I mention this because in every book you are told that dirt and worms are two chief causes of it. Now I do not believe it, because dirt is very common, and I have found no reason in my experience for thinking that dirt produces it more frequently than cleanliness. In the same way it is said to be due to worms. Now, while I have seen many cases of worms without it, I have never seen a case of vulvitis that I could ascribe to worms. And I believe that this is an illustration of the injurious tendency to repeat what has been said before. Because one author of repute says a thing every one repeats it. Every one of you has been taught that worms cause convulsions in children, but were I lecturing on the subject of convulsions I should make the same skepitcal remarks on that head. I never saw a case of convulsions that I could reasonably trace to worms, and I never saw a case of worms that caused convulsions.—*Prof. J. Mathews Duncan, in Med. Times and Gaz.*

